# Data set on prediction of friction stir welding parameters to achieve maximum strength of AA2014-T6 aluminium alloy joints

**DOI:** 10.1016/j.dib.2019.103735

**Published:** 2019-03-14

**Authors:** C. Rajendran, K. Srinivasan, V. Balasubramanian, H. Balaji, P. Selvaraj

**Affiliations:** a Department of Mechanical Engineering, Sri Krishna College of Engineering and Technology, Coimbatore, Tamilnadu, 641008, India; b Center for Materials & Joining and Research (CEMAJOR), Annamalai University, Tamilnadu, India; c Aeronautical Development Agency, Bangalore, Karnataka, India

## Abstract

Statistical tools such as design of experiments (DoE), analysis of variance (ANOVA) were used to develop the empirical relationship, to predict the ultimate tensile strength of the joint at the 95% percent confidence level. Response surface graph and contour plots were constructed using response surface methodology (RSM) concept. From this investigation, it is found that the joint fabricated with a tool rotational speed of 1500 rpm, welding speed of 40 mm/min, tool tilt angle of 1.5° and tool shoulder diameter of 6 mm, exhibited maximum tensile strength of 380 MPa.

**Table:** Specifications table

Subject area	*Materials science and Engineering*
More specific subject area	*Solid state welding - Friction stir welding*
Type of data	*Tables, Figures, Text*
How data was acquired	*Macrostructure analysis was performed using a stereo-zoom macro scope (Make: Macro scope Z: Model CM 0646). Microstructure analysis was carried out using light optical microscope (Make: MEIJI, Japan, Model: ML7100). Tensile strength was evaluated using universal testing machine (Make: FIE-BLUE STAR, India; Model: UNITEK-94100). Response surface graph was drawn using a design expert software. v 8.1.*
Data format	*Raw, analyzed.*
Experimental factors	2 mm *thick AA2014-T6 aluminium alloys sheets were used in this investigation with chemical composition of 4.2% Cu, 0.6% Mn and 0.4%Mg. Computer numerical controlled friction stir welding(FSW) machine was used to fabricate the joints. The tool was made of super HSS with pin diameter of* 2 mm*. Four factors five level central composite design matrix were used to establish empirical relationship to predict tensile strength of FSW joints.*
Experimental features	*The response surface methodology (RSM) was used to predict optimum tensile strength of friction stir welded butt joints of AA2014 aluminum alloy. The adequacy of the developed model was found using ANOVA.*
Data source location	*Center for Materials Joining & Research, Annamalai University, Tamil Nadu, India*
Data accessibility	*The data with this article.*
Related research article	*C.Rajendran, Srinivasan, V. Balasubramanian, H. Balaji, P. Selvaraj. Identifying combination of friction stir welding parameters to maximize strength of lap joints of AA2014-T6 aluminium alloy, Australian Jour. of Mechanical Engg., 2017,**https://doi.org/10.1080/14484846.2017.1304843**.*

**Table:** 

**Value of the data** • Design experiments is a concise tool to reduce no of trail runs in process parameters optimization. • Response surface methodology is an efficient tool to optimize process parameters. • Analysis of variance (ANOVA) is a stastical tool to find weight of each process parameters and significance of developed model. • Response surface graph can give visual data to find maximum response

## 1 Data

**Fig. 1:**
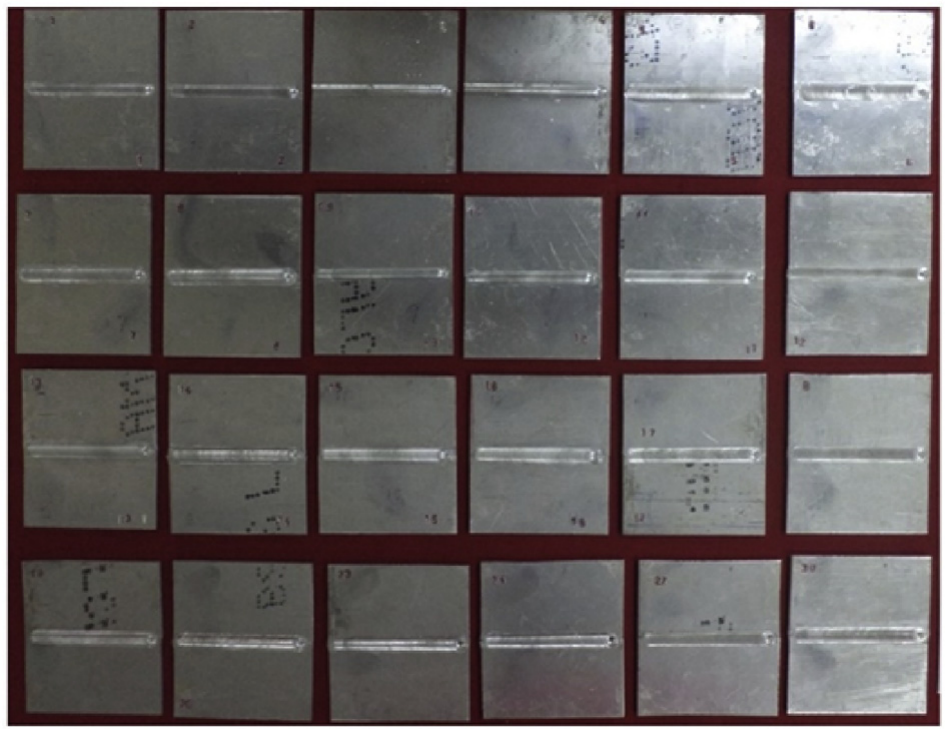
Photograph of fabricated FSW joints.

**Fig. 2:**
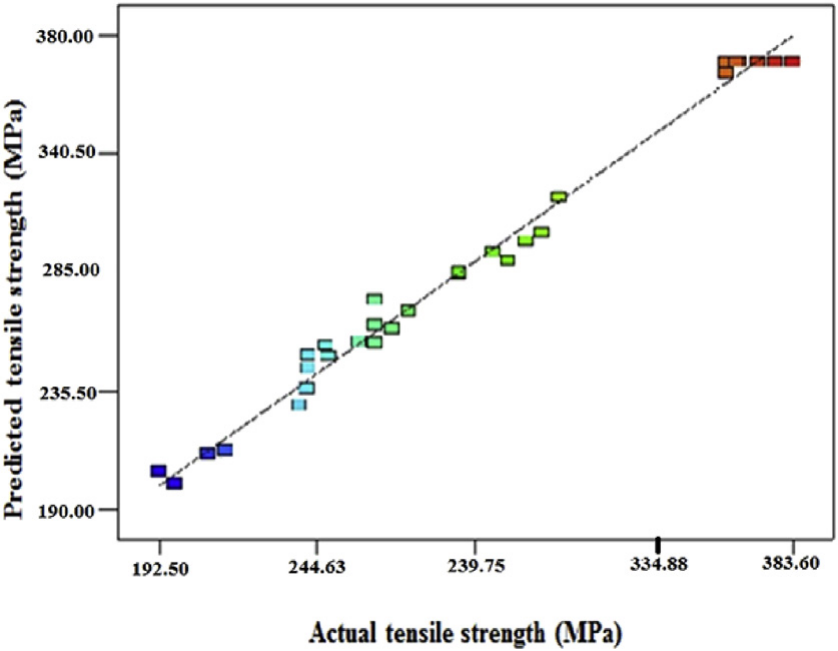
Actual Vs predicted tensile strength

**Fig. 3:**
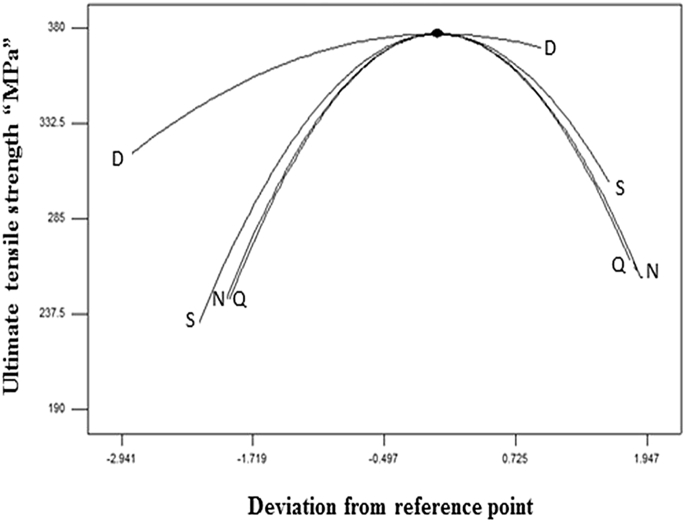
Perturbation graph.

**Fig. 4:**
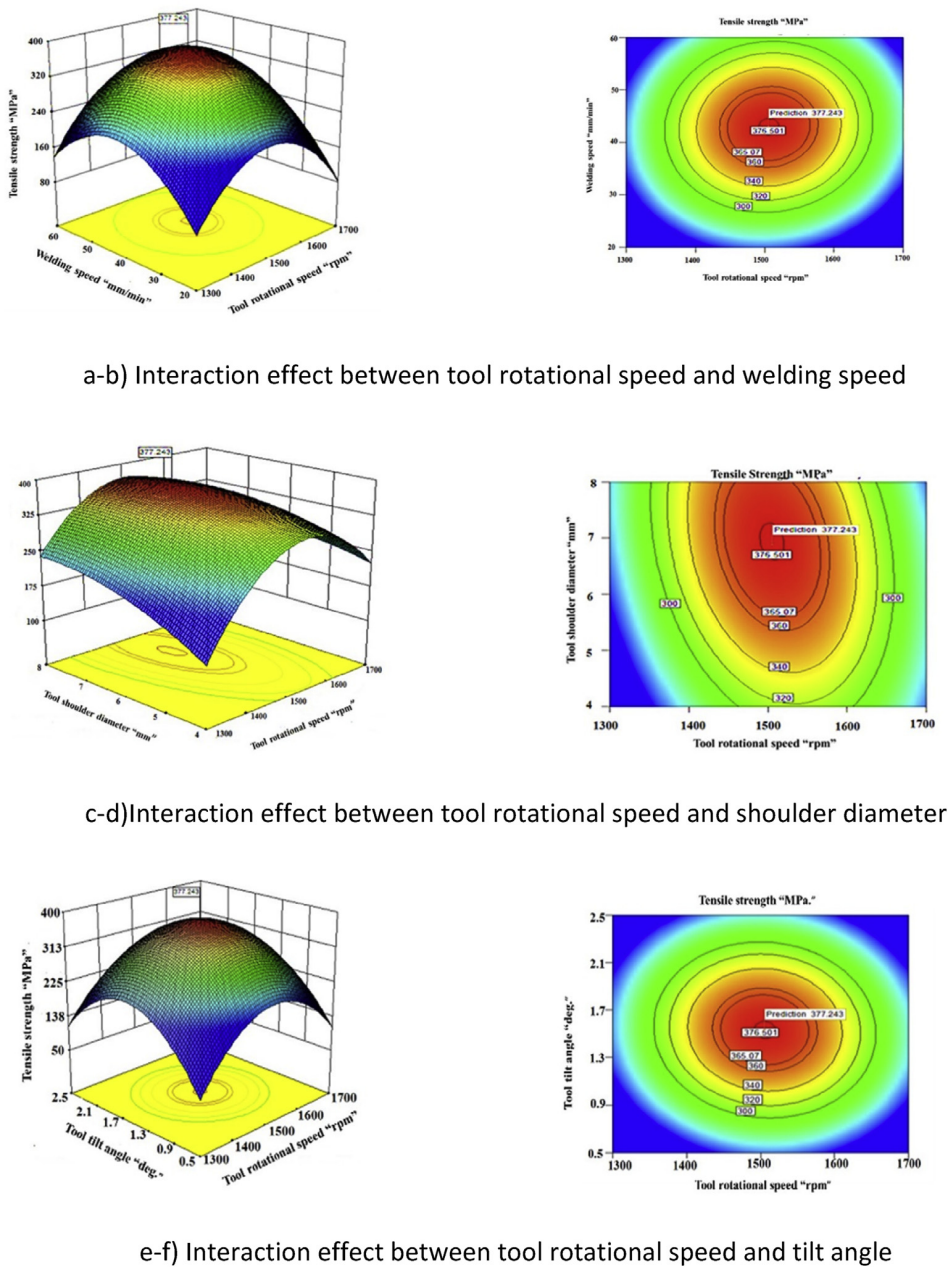

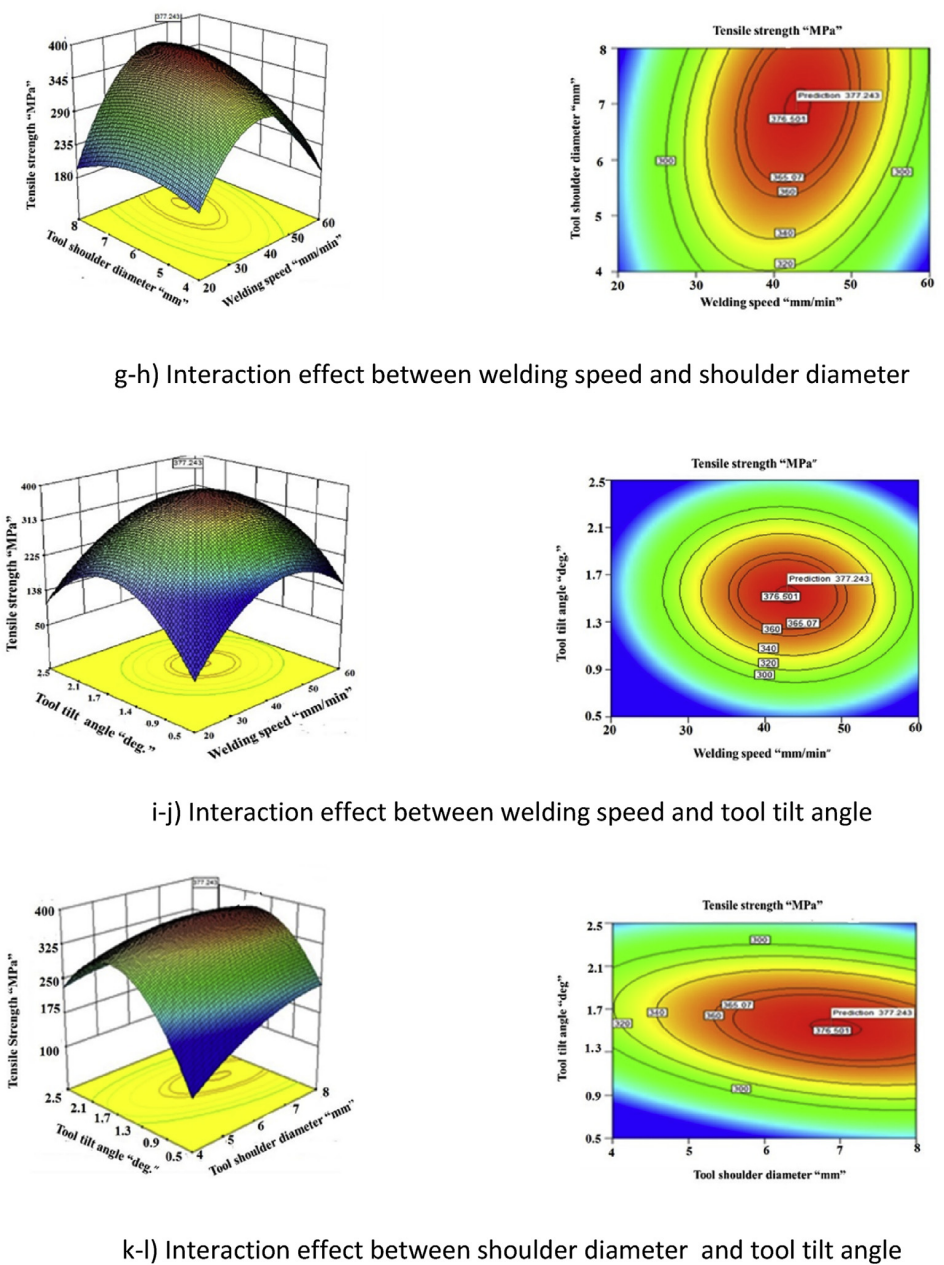
Response surface graph.

The data presented in this paper illustrate optimizing friction stir welding parameters to attain maximum strength of AA2014-T6 aluminium alloy. The following parameters were used such as tool rotational speed (N), welding speed (S), shoulder diameter (D) and tool tilt angle (Q) [1], [2]. Data for fixing feasible working range of each parameters are presented in Table 1. FSW parameters and its working range are presented in Table 2. Design matrix and its data, calculated data of co efficient, ANOVA data, validation data are presented in Table 3, Table 4, Table 5, Table 6 respectively. Fig. 1 represented fabricated joints using experimental data. Fig. 2 shows predicted and actual data. The perturbation data are provided in Fig. 3 and Effect of process parameters and its response as shown in Fig. 4.

**Table 1:** Macrostructure analysis for fixing the working range of FSW.

Process parameters	Parameters range	Macrograph	Name of the defect	Reason for defect
Tool rotational speed (N)	N> 1700 rpm		Cluster of worm hole	Excess heat input
Tool rotational speed (N)	N<1300 rpm		Lack of fill	Insufficient heat input causes less plastic material flow
Welding speed (S)	S>60 mm/min		Tunnel defect	Low plasticized material transportation
Welding speed (S)	S<20 mm/min		Warm hole	High heat input produced
Tool shoulder diameter (D)	D>8 mm		Cluster of worm holes	Excess heat input due to large area of contact
Tool shoulder diameter (D)	D<4 mm		Tunnel defect	Low heat generation produced insufficient plasticized material transportation
Tool tilt angle (Q)	Q>2.5		Cluster of warm hole	High forging pressure produced more strain hardening
Tool tilt angle (Q)	Q<0.5		Lack of fill	Insufficient forging force resulted low plasticized material flow and consolidation

**Table 2:** FSW parameters and their working range.

Sl.No	Parameters	Unit	Notation	Levels				
				−2	−1	0	+1	+2
1	Tool rotational speed	rpm	N	1300	1400	1500	1600	1700
2.	Welding speed	mm/min	S	20	30	40	50	60
3	Tool shoulder diameter	mm	D	4	5	6	7	8
4	Tool tilt angle	deg.	Q	0.5	1.0	1.5	2.0	2.5

**Table 3:** Design matrix and experimental results.

S Expt. no	Coded values				Actual values				Tensile strength (MPa)
	N	S	D	Q	N “rpm”	S “mm/min”	D “mm”	Q “deg.”	“MPa”
1	−1	−1	−1	−1	1400	30	5	1	200
2	+1	−1	−1	−1	1600	30	5	1	242
3	-1	+1	-1	−1	1400	50	5	1	214
4	+1	+1	−1	−1	1600	50	5	1	257
5	−1	−1	+1	−1	1400	30	7	1	242
6	+1	−1	+1	−1	1600	30	7	1	248
7	−1	+1	+1	−1	1400	50	7	1	285
8	+1	+1	+1	−1	1600	50	7	1	309
9	−1	−1	−1	+1	1400	30	5	2	261
10	+1	−1	−1	+1	1600	30	5	2	285
11	−1	+1	−1	+1	1400	50	5	2	261
12	+1	+1	−1	+1	1600	50	5	2	299
13	−1	−1	+1	+1	1400	30	7	2	270
14	+1	−1	+1	+1	1600	30	7	2	266
15	−1	+1	+1	+1	1400	50	7	2	295
16	+1	+1	+1	+1	1600	50	7	2	304
17	−2	0	0	0	1300	40	6	1.5	218
18	+2	0	0	0	1700	40	6	1.5	242
19	0	−2	0	0	1500	20	6	1.5	239
20	0	+2	−2	0	1500	60	6	1.5	261
21	0	0	+2	0	1500	40	4	1.5	314
22	0	0	0	−2	1500	40	8	1.5	361
23	0	0	0	+2	1500	40	6	0.5	204
24	0	0	0	0	1500	40	6	2.5	247
25	0	0	0	0	1500	40	6	1.5	380
26	0	0	0	0	1500	40	6	1.5	370
27	0	0	0	0	1500	40	6	1.5	375
28	0	0	0	0	1500	40	6	1.5	361
29	0	0	0	0	1500	40	6	1.5	365
30	0	0	0	0	1500	40	6	1.5	364

**Table 4:** Calculated values of coefficients.

Coefficient	Factor estimate
Intercept	369.17
N (rpm)	9.58
S (mm/min)	10.58
D (mm)	12.25
Q (deg.)	13.75
NS	2.88 (Not significant)
ND	−7.00
NQ	−3.00 (Not significant)
SD	7.75
SQ	−3.5 (Not significant)
DQ	−8.87
N^2^	−34.1
S^2^	−29.1
D^2^	−7.23
Q^2^	−35.23

**Table 5:** ANOVA test results.

Source	Sum of Squares	df	Mean Square	F Value	p-value	
					Prob > F	
Model	86491.46	14	6177.96	91.61	<0.0001	significant
N	2204.16	1	2204.16	32.68	<0.0001	significant
S	2688.16	1	2688.16	39.86	<0.0001	significant
D	3601.5	1	3601.5	53.4	<0.0001	significant
Q	4537.5	1	4537.5	67.28	<0.0001	significant
NS	132.25	1	132.25	1.96	0.1817	
ND	784	1	784	11.62	0.0039	significant
NQ	144	1	144	2.13	0.1646	
SD	961	1	961	14.25	0.0018	significant
SQ	196	1	196	2.9	0.1088	
DQ	1260.25	1	1260.25	18.68	0.0006	significant
N^2^	31902.01	1	31902.01	473.08	<0.0001	significant
S^2^	23233.44	1	23233.44	344.53	<0.0001	significant
D^2^	1433.44	1	1433.44	21.257	0.0003	significant
Q^2^	34041.44	1	34041.44	504.81	<0.0001	significant
Residual	1011.5	15	67.43			
Lack of fit	748.66	10	74.86	1.42	0.3654	Not significant
Pure error	262.83	5	52.56			
Cor. Total	87502.96	29

**Table 6:** Validation test results for developed empirical relationship.

Sl. no	Tool rotational speed (rpm)	Welding speed (mm/min)	Tool shoulder diameter (mm)	Tool tilt angle (◦)	Actual TS (MPa)	Predicted TS (MPa)	Variation
01	1500	50	6	1.5	360.0	364.0	−4.0
02	1450	60	7	1.5	284.0	289.0	−5.0
03	1550	40	6	1.5	364.5	359.0	+5.0

## 2 Experimental design, materials, and methods

### 2.1 Feasible working limit of FSW parameters

Different combinations of FSW parameters were used to carry out the trial experiments. This was done by changing any one of the factors from minimum to maximum, while keeping the other parameter at constant values (Table 1).

The feasible working limits of the individual parameters were identified by inspecting tunnel, lack of fill, warm holes’ defects, top surface of the weld, macrostructure for a smooth appearance without any visible macro level defects such as pinhole and root defect. The chosen levels of the selected process parameters are presented in Table 2.

### 2.2 FSW experiments and UTS evaluation

The FSW joints were fabricated as per the conditions dictated by the design matrix (Table 3) at random order [2], [3]. A tool with a ﬂat concave shoulder and tapered pin were used in FSW. A computer numerical controlled FSW machine was used to fabricate the joints. At each condition, three specimens were fabricated and some of the fabricated FSW joints are displayed in Fig. 1. The data of tensile strength were recorded and presented in Table 3. The RSM has been used to predict the maximum tensile strength [4] of butt joints of AA2014 aluminum alloy in terms of the important FSW parameters.

### 2.3 Developing a mathematical relationship

(1) Tensile Strength of FSW joint = f (N, S, D, Q)

The significance of each co-efficient was calculated from student t-test and p-values, which are listed in Table 4, The final empirical relationship was constructed using only these co-efficient [5], [6] and the developed empirical relationship of FSW joints is given below

(2) UTS = [+369.16+9.58 (N)+10.58 (S)+12.25(D)+13.75(Q)-7.0(ND) +7.75 (S D)– 8.87(D Q)-34.1 (N^2^) -29.1(S^2^) -7.2(D^2^)- 35.22 (Q^2^) MPa

The adequacy of the developed model is tested by ANOVA. The test results of the ANOVA are given in Table 5; the desired confidence level was 95%. The relationship may be considered to be adequate. Fig. 2 shows the correlation graph of predicted and actual tensile strength of FSW joints, it could indicate the deviation between the actual and predicted UTS is low. Each predicted data matches with the experimental data is well shown in Fig. 2.

The Fisher’s F -test with a very low probability value demonstrates a very high significance of the regression model. The goodness of fit of the model is fitted by the determination coefficient (R^2^). The coefficient of determination was calculated to be 0.9884 in response which implies that 98.8% of the experimental values confirm the compatibility with data as predicted by the model. Fig. 3 illustrates the perturbation plot for the response tensile strength of FSW joints.

### 2.4 Optimizing FSW parameters

By analyzing the response surface and contour plots as shown in Fig. 4(a–k), the maximum achievable tensile strength is found to be 377.21 MPa. The corresponding parameters that yield this maximum value are tool rotational speed of 1505 rpm, welding speed of 43.08 mm/min, tool shoulder of 6.95 mm and tool tilt angle of 1.53°. The higher F ratio value implies that the respective levels are more significant.
