# Supplementary material for: Data set on prediction of friction stir welding parameters to achieve maximum strength of AA2014-T6 aluminium alloy joints
Source: Data Brief. 2019 Mar 14;23:103735. doi: 10.1016/j.dib.2019.103735 (PMC6661257; doi:10.1016/j.dib.2019.103735)
Supplement: Multimedia component 1 [file mmc1.docx]

Conflict of Interest Form

Coimbatore

27.1.2019

To

The Editor- In-Chief,

Data in Brief.

Respected Sir,

Ref. i)Title: Dataset on prediction of friction stir welding parameters to maximize tensile strength of AA2014-T6 aluminium alloy

ii) DIB No: DIB-D-18-02996R1

iii) DIB-D-18-02996R2

iv) Authors: C. Rajendran, Srinivasan, V. Balasubramanian, H. Balaji, P. Selvaraj

Warm greetings, I happy to submit my revised manuscript with properly addressed. Whichever asked by the editor. On behalf of all authors, I assured that we have no conflict. Hence I request you to proceed for further process.

Thanking You

C.Rajendran
